# Borderland Economic Resilience under COVID-19: Evidence from China–Russia Border Regions

**DOI:** 10.3390/ijerph192013042

**Published:** 2022-10-11

**Authors:** Yuxin Li, Pingyu Zhang, Kevin Lo, Juntao Tan, Qifeng Yang

**Affiliations:** 1 Northeast Institute of Geography and Agroecology, Chinese Academy of Sciences, Changchun 130102, China; 2 College of Resources and Environment, University of Chinese Academy of Sciences, Beijing 100049, China; 3 Department of Geography, Hong Kong Baptist University, Kowloon Tong, Hong Kong 999077, China; 4 School of Geography, Geomatics and Planning & Urban-Rural Integration Development Research Institute, Jiangsu Normal University, Xuzhou 221116, China

**Keywords:** economic resilience, COVID-19, border region, spatial differentiation, determinants, China–Russia border

## Abstract

The COVID-19 pandemic has had a great impact on the global economy and trade, and border regions have been hit severely because of their high dependency on foreign trade. To understand better the economic impact of COVID-19 on border regions, we developed a COVID-19 economic resilience analytical framework and empirically examined 10 Chinese-Russian border cities in Northeast China. We quantitatively analyzed five dimensions of economic resilience, distinguished four types of shock, and examined the determinants of economic resilience. The results show that: (1) the COVID-19 pandemic has wide-ranging impacts in the border areas, with import–export trade and retail sales of consumer goods being the most vulnerable and sensitive to the shock. The whole economy of the border areas is in the downward stage of the resistance period; (2) from a multi-dimensional perspective, foreign trade and consumption are the most vulnerable components of the borderland economic system, while industrial resilience and income resilience have improved against the trend, showing that they have good crisis resistance; (3) borderland economic resilience is a spatially heterogeneous phenomenon, with each border city showing different characteristics; (4) economic openness, fiscal expenditure, and asset investment are the key drivers of economic resilience, and the interaction between the influencing factors presents a nonlinear and bi-factor enhancement of them. The findings shed light on how border economies can respond to COVID-19, and how they are useful in formulating policies to respond to the crisis.

## 1. Introduction

The COVID-19 pandemic has created global economic risks and uncertainties, and it has disrupted the global trade networks [1,2,3] and border regions as the main portal of foreign trade and economic openings are at the forefront of the pandemic-induced economic shock. Furthermore, most of the border regions have been remote, peripheral, and highly dependent on a single mode of economic activity (e.g., trade), making their economies more vulnerable to external disturbances and less capable of swiftly adapting to shocks [4,5,6].

Yet, the economic impact of the COVID-19 pandemic on the border regions has yet to be examined either theoretically or empirically as most studies on the borderlands and COVID-19 have instead focused on health, border control, and cross-border cooperation [7,8,9]. This study aims to understand such dynamics from the theoretical lens of regional economic resilience. The notion of resilience has become popular not only in disciplines like physics, ecology, and disaster science, but also in economics, regional studies, and geography. It provides a new perspective to explain the recession, adaptation, and transformation of the regional responses to the crisis [10,11]. Especially after the 2008 global financial crisis, many economic and urban geographers have devoted much attention to the theoretical and empirical advancement of regional economic resilience to explain why economies differ in their recovery from common challenges and what determines regional resilience in an uncertain context [12,13,14,15].

With a complex regional system of human–land relationships, these regions are often affected by various disturbances and shocks [16]. Martin and Sunley [10] point out that the nature of risk (source, type, and duration) is closely linked to regional resilience. The outbreak of COVID-19 at the end of 2019 had a catastrophic impact on the global economy. Limited by the epidemic control measures, the flow of the production materials and population were blocked, and all of the sectors of the industrial division system struggled to operate normally [17]. At the same time, the consumer market was depressed, and import and export trade mechanism was blocked, which in turn affected the global, national, and regional industrial chains [18]. As a result, the crisis not only had a strong impact on the open coastal regions with a high degree of economic specialization and diversification, but also on the border regions with a weak financial system and a high degree of external dependence. In the context of a large global economic recession, the impact of COVID-19 also had a significant spatial differentiation. According to evolutionary economic geography, regional economic resilience refers to the ability of regional economy to recover, renew, and transform itself through re-organization under the external disturbance [10]. As such, it can provide a key insight into the unique evolutionary mechanisms that function in border regions. In the context of globalization, border areas are not only frontiers for opening-up and cooperation, but they are also high-risk regions that were affected by COVID-19. Therefore, understanding how the border areas responded and adapted to the COVID-19 crisis, and to seek avenues of recovery in the post-pandemic era is of pertinence to the regional economic resilience.

Empirically, this study focuses on the China–Russia border regions in Northeast China. China’s northeast border areas are the heartland of the Northeast Asia Economic Circle and the Pacific Rim Economic Belt. They have played very crucial roles in China’s opening-up and international economic cycle. It was predicted that at the critical stage of high-quality development in China’s border regions, the outbreak of COVID-19 would have had a significant impact on the macro economy, meso industries, and micro individuals in the short term [19]. From a macroeconomic perspective, the pandemic brought the obstruction of the cross-border flow of labors, capital, and goods, thereby resulting in a sharp reduction in the production and trade capacity, as well as supply shortages and price increases [20]. From the perspective of the meso-level industries, the pandemic severely hit the key sectors of the tertiary industry in border regions, such as tourism, catering, retail, and logistics, which limited the market consumer demand. From the micro individual perspective, the interruption of personal income which was caused by the shutdown of the enterprises further brought a double-blow to the production and consumption market. Therefore, the economic resilience of the border regions needs to be interpreted in terms of the multi-dimensional indicators such as the GDP, import and export trade, industry, income, and consumption. At the same time, the economy of China’s border regions has long relied on border trade and resource-based industries. In the context COVID-19, the high external dependence and single industrial structure made the border regions easily disturbed by the external shocks (COVID-19), which led to the interruption of the regional development path and a fall into the track of recession. In the strategic context of the “Belt and Road Initiative” and the construction of the “China-Russia-Mongolia Economic Corridor”, how to cope with the impact of the uncertainties has become the key to maintaining the high-quality and stable development of the border regions. Therefore, there is an urgent need to study the resilience characteristics of the border regions in response to COVID-19, especially to explore the influencing mechanisms of the evolution of import and export trade, industry, income, and consumption resilience in the macro to micro scales. This is of great significance for improving the ability of the border regions to cope with the external shocks and maintain a sustainable development path.

Therefore, this paper tries to make some contributions to the study of regional economic resilience from two aspects: the measure of regional economic resilience and the spatial heterogeneity of the influence mechanism. By creating a resilience-based analytical framework and collecting the quantitative evidence from the prefecture-level border cities in Northeast China that are adjacent to Far East Russia, this paper aims to reveal the spatial characteristics and determinants of the borderland economic resilience amidst the COVID-19 pandemic. More specifically, this study attempts to answer the following three questions: (1) What were the specificities of the border regions’ economic systems when they were coping with the COVID-19 pandemic? (2) Do the economic resilience mechanisms of the Sino-Russia border regions show the same geographies in their responses to the crisis? (3) What determinants shape a higher degree of urban economic resilience? The remainder of this research is organized as follows. Based on the literature review on the definition, evaluation, and mechanism detection of regional economic resilience, Section 2 provides a framework for the economic resilience in the border areas. Section 3 introduces the study area, methods, and data sources. After presenting the main results in Section 4, Section 5 summarizes the major conclusions.

## 2. Literature Review and Theoretical Framework

### 2.1. Economic Characteristics of Border Regions

The current urban classification system mainly relies on the population scale and functional characteristics, while the border regions are defined according to their location. The concept of “border” in the modern sense refers to the territory, high seas, and airspace of a country within the border line [21]. In this paper, the “border region” refers to the area with a national border that is adjacent to that of another country/countries. In the present era, the connotation of a border is not only a linear, but it is a comprehensive place with national power, and an economic, social, and ethnic living space [22]. Especially in the context of the global economic network, the border regions have evolved from the traditional “marginal area” of security to the “core area” of multiple functions including territorial security, economic trade, cultural exchange, and livelihood security. Furthermore, the political economy of the border regions is more complicated, as the political situation, trade environment, and policy preferences of the neighboring countries are of crucial importance to border regions [23].

In the context of globalization, border regions are not only the boundary of safeguarding national territorial sovereignty, but also the frontier of interconnection with the neighboring nations, serving as the open gateway and connection link for the cross-border flow of population, commodities, resources, and information [22]. However, as they are the core catalyst for the interaction with neighboring countries, border regions are significantly affected by anti-globalization forces, as the global financial crisis, local wars, and the outbreak of diseases like the COVID-19 pandemic make the global geopolitical environment increasingly complex, and the border areas become the tensest and most sensitive regions for foreign relations. Furthermore, border regions are generally home to poor minority populations, so there have been many problems such as population loss, industry disruption, security threats, and a lower degree of openness. At the same time, many border ports do not play a prominent role in driving the cities’ development, and the phenomenon of “cargo channels” is significant [24], meaning that these areas have lower capacities to participate in international competition, gradually becoming “islands” of administrative management and economic development.

The development of China’s border regions has profound institutional, cultural, and economic motivations. Since China implemented the border opening strategy in 1992, the border regions have always been the open door and cross-border gateway for cooperation between China and its neighboring countries. Through the implementation of border opening-up policies, state agencies have played a leading role in expanding neighboring markets and utilizing external resources [23], thus transforming border areas into hubs for border trade, cross-border commerce, industry cooperation, and cultural communication. Although some regional policies have accelerated the growth process of the border regions, their sustainable development is still facing serious challenges. Compared with other inland cities in China, due to factors such as natural environment, traffic conditions, industrial bases, and geography, the economic development of China’s border regions tend to be relatively weak, and the urbanization process remains slow [25]. Meanwhile, compared with coastal open cities, the national security role of the border regions often dominates their economics, which further presents challenges to their economic development [26].

Since 1992, when the State Council designated 14 cities, such as Manzhouli, Suifenhe, and Hunchun, as border open cities, trade activities such as dweller trade, barter trade, and small-scale trade have gradually been carried out with neighboring Russia [27], which plays a special strategic role in promoting border stability and prosperity. However, with the geographical situation in Northeast Asia and the corresponding situation in the market in Russia, the trade function of the Sino-Russian border area is insufficient, and its port infrastructure is vulnerable, thus forming a so-called “double-periphery” of geographical location and macroeconomy. Moreover, the similarity between the geographical positions and the natural conditions endows the border regions with some commonalities: traditional border trades and port services (food, clothing, housing, and transportation) occupy a large proportion of the economy, and the mode and function of the ports’ development tend to be similar, lacking a reasonable and distinct regional division. Meanwhile, the border ports are large in number but small in scale, lacking coordinating linkages with the inland hinterland, and the competition for hinterland resources hampers the regional coordination and development [27]. Furthermore, a population loss is caused by an uneven regional development is unidirectional, long-term, and large-scale, which not only leads to a drop in demographic dividends, but also impacts national defense [28]. Finally, the environmental protection and the city construction lack coordinated action, and the resources are lacking to support the economic and social development.

### 2.2. Theorizing Economic Resilience for Border Regions amidst the COVID-19 Pandemic

The interest in regional economic resilience stems from the realization that different economies have great heterogeneity in their ability to cope with the current crisis. Some of the regions can shake off the difficulties that are brought about by the crisis and realize economic transformation and upgrading; while some regions may enter the track of long-term decline [12]. The concept is linked to a group of related concepts including “engineering resilience,” “ecological resilience,” and “evolutionary resilience” [29,30,31,32]. Among these different conceptual linkages, evolutionary resilience has been most influential in explaining regional economic resilience [33]. Although there is no universally accepted definition, scholars generally agree that regional economic resilience reflects the process of path dependence and path creation through continuous adjustment and adaptation [13,29,34]. Common features of this include the following three aspects:

1. Being affected by a disturbance or stressors—including sudden shock (earthquake, flood, financial crisis, and a COVID-19 pandemic) and slow-burn decline (population shrinkage, industrial decline, and economic recession).
2. The ability to maintain, repair, and quickly return to regional system stability.
3. The ability to successfully resist internal and external disturbances or stressors, and recover, adapt, and reorganize the regional structure from these negative influences.

Based on the comprehensive features that are noted above, this paper holds that the region is a complex adaptive system. As an inherent attribute of the region, economic resilience refers to the ability of the regional economic system to resist a disturbance and recover from it, or to realize a path breakthrough through adjustment and transformation to create renewed development. Its coping ability not only relates to the short-term shocks but also its long-term ability to get rid of its dependence on this path and develop new growth paths. Therefore, regional economic resilience is the ability of a regional economy to actively adjust and transform itself in response to short-term shocks and long-term changes in the development process, and it represents the adaptability, innovation, and sustainability of the region. The sustainability theory of a regional economy emphasizes the systematic nature of the regional economic development and coordination [35], and it focuses on whether the regional economic system can develop healthily, rationally, and continuously, which coincides with the concept of “resilience for what purpose” in the regional resilience theory. Therefore, the identification of regional economic resilience is a new direction for geographical sustainable development research. Within this, the identification of regional economic resilience is the basis of it, the assessment of regional economic resilience is the means of it, and the enhancement of regional economic resilience and a sustainable development ability is the goal of it [36].

A growing number of scholars have acknowledged that regional economic systems are subject to a shock-prone process [37]. Actors and agencies need to make short-term and long-term preparations to adapt to frequent challenges. In addition, research has shown that resilience is not about something you have, but it is more about the ways in which you react [16], which underlines the influence of regional preconditions and an agency ability on regional economic evolution. Therefore, scholars have gradually combined evolutionary resilience with existing theories such as “path-dependence,” “path-breaking,” “locking and unlocking” to study regional economic resilience [13,38].

Based on the adaptive cycle model [29,39], the resilience of the regional economic system involves four dimensions. The first one is resistance, which mainly refers to the vulnerability or sensitivity of the regional economy in the face of shocks that occur, manifesting as the decline in regional GDP, industrial output value, employment rate, and other economic indicators over a short period. The second process is recovery, which mainly reflects the speed and degree of the regional economic recovery. The third one is reorganization, which refers to the ability of regional economic systems to actively adapt and restructure themselves in response to the shocks, such as the adjustment and transformation of the industrial structures, technological levels, and business models. The fourth process is renewal, which refers to the ability of regional economic systems to update their original development path and create new growth paths for “bouncing forward” [10]. These four dimensions reflect the different development stages of regional economic resilience. Meanwhile, in the process of regional evolution, these dimensions interact with each other and jointly constitute the expression of regional economic resilience.

The assessment methods of economic resilience mainly include a case analysis, a resilience index, a time series model, and a causal structure model [10]. They may take the 2008 financial crisis as a short-term sudden shock [40], or the change in the national economic cycle as a long-term slow burn disturbance [41,42]. From the perspective of the influencing mechanisms, many scholars have discussed the effects of the economic scale, industrial structure, network relations, and location conditions on regional economic resilience [10,43,44]. Studies have generally found that a larger economic scale, advanced production, and technological innovation improve the regional economic resilience. However, there are some different understandings about the functions of industrial structures, social assets, economic openness, and public policies [15,38,45,46,47].

The nature of risks is key to regional resilience [10]. The source, type, scale, and target of the shocks, as well as local socio-economic conditions, are of crucial importance to understanding regional economic resilience. The intensity and duration of these crises are closely related to the type of resilience that there is. Low-intensity and short-duration ones may lead to bounce-back resilience; while a high-strength and long-duration shock may contribute to transformation resilience [14,48]. Moreover, even if the shock is a one-off event, it may produce a long-lasting and profound effect on the regional economic system [37].

The COVID-19 pandemic is a truly global crisis, and it has brought about the largest and deepest economic recession since the Great Depression [1]. Due to the public health crisis, regional blockades, border controls, factory shutdowns, and social contact restrictions have had profound consequences for the production and consumption in cities. Relevant studies have stressed that regions with a higher economic openness can enhance their local economic strength by globally interconnecting with other regions. However, the validity of this statement largely depends on the nature and scope of the crisis. If the crisis restricts the international logistics or import and export trades, then the more closely connected they will be with the world, and therefore, the more vulnerable and sensitive the region will be [14].

Studies have also emphasized that the new (short-term) shock is related to the existing (long-term) slow-burn shock, and that the new shock can aggravate or weaken the influence of a long-term disturbance [49], thus bringing about the mid-term structural shocks with cumulative effects [50]. Therefore, regional economic resilience in dealing with short-term shocks such as the COVID-19 pandemic is also affected by local long-standing, deep-seated structural problems. At the same time, the challenges that have been brought about by the crisis will also help the region to create new development paths, learn new coping experiences, and meet new development needs. Although the COVID-19 pandemic increases the threat of infection, interrupts the production processes, and negatively affects the international supply chains, it also promotes the structural adjustments in cross-border e-commerce, international logistics, industrial cooperation, and port functions, which constitute the characteristics of regional long-term transformation.

To sum up, the regional economy is a highly complex resilience system. As a special region, the border regions have long-standing endogenous problems in terms of their economic structure, institutional mechanisms, and innovation ability. Therefore, fluctuations in the economic resilience of the border area are reflected in the impact of sudden shocks (COVID-19) under processes of long-term structural evolution. Since the pandemic is still ongoing at the time of us writing this paper, according to the resilience classification process by Martin et al. [44], the current economic resilience of the border regions is primarily related to their vulnerability and resistance, which is accompanied by a partial recovery. Therefore, from a short-term perspective, this paper particularly explores the ability of border regions to resist and recover from the COVID-19 pandemic in the context of the endogenous problems of it.

**Figure 1:**
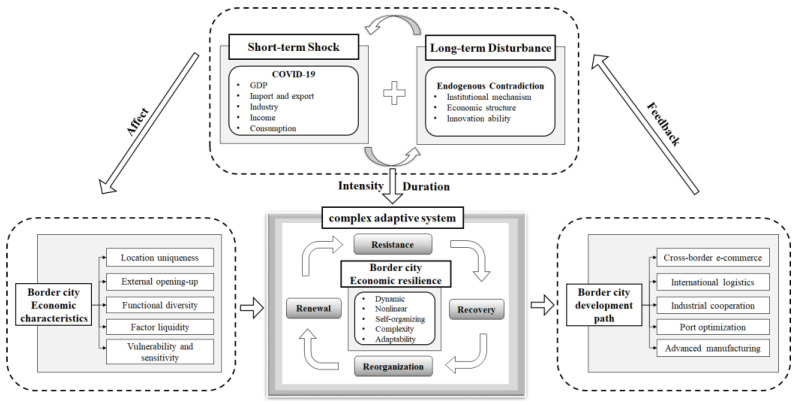
Theoretical framework: borderland economic resilience during the COVID-19 pandemic.

The existing mainstream studies on resilience mainly use GDP as a crucial indicator of regional economic resilience [51,52]. This study argues that the indicators of regional economic resilience need to expand from a single dimension to multiple dimensions. At the same time, the socio-economic problems that have been caused by COVID-19 in the border regions should not only focus on the production side but also include indicators from the consumption side [53]. As border regions are dominated by a market-led and export-oriented economy, foreign trade and investment are the carriers and representations of a geo-economic system. When a global crisis occurs, the demand for international products declines, and the import–export trade is the most vulnerable element of this. The disruption of foreign trade further hits global, national, and regional industrial chains. Meanwhile, the use of epidemic prevention and strict measures restricts the flow of production means and capital information, making it difficult for industrial enterprises to resume work and weakening their output value [54]. If the crisis continues, then the regional economy will be in recession, thus leading to there being less income for the public. Moreover, epidemic control policies and the reduction in the expected income generate a further downturn in consumer demand. Therefore, in this paper, GDP was selected to be the key indicator to evaluate the economic resilience of the entire region. The total import–export trade value, the industrial added value above scale, the per-capita disposable income of the urban residents, and the total retail sales of social consumer goods were selected to represent the production and consumption dimensions. By comparing the variations in the five resilience indicators in the border area from 2019 to 2020, we can more accurately capture the economic resilience characteristics that were affected by the COVID-19 pandemic. The theoretical framework is shown in Figure 1.

## 3. Methods and Data

### 3.1. Study Area

China is located in the center of Asia, with a total land border of 22,000 km, and it is adjacent to 14 countries, including Russia, Mongolia, Kazakhstan, India, and Myanmar. Among them, the Sino-Russian borderline was demarcated by the two countries on 14 October 2004, and it has a length of 4319.78 km, accounting for about 1/5 of China’s borderline. The boundary rivers include the Argun River, Heilongjiang River, Ussuri River, the Songatcha River, and Xingkai Lake, totaling about 2300 km [26].

**Figure 2:**
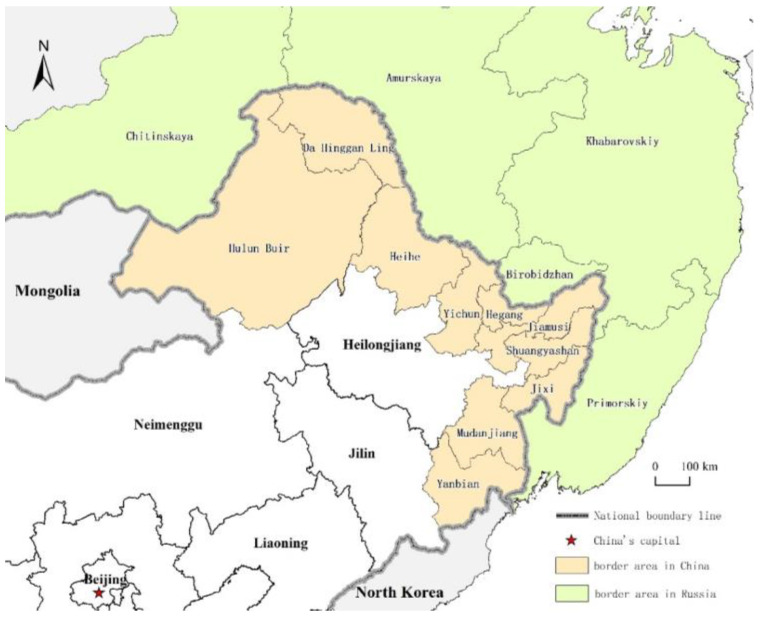
Location of Sino-Russian border area.

The study area of this paper is China’s border area which is adjacent to Far East Russia. The border area refers to the collection of administrative units in the prefecture-level cities (autonomous prefecture, league) of Heilongjiang, Jilin and eastern Inner Mongolia, China, which border Russia along the national boundary [26], including Heihe, Yichun, Hegang, Jiamusi, Shuangyashan, Jixi, Mudanjiang, and the Da Hinggan Ling prefecture in Heilongjiang, the Yanbian Korean autonomous prefecture in Jilin (Yanbian for short), and Hulun Buir in Inner Mongolia, totaling 10 border cities on the Chinese side (Figure 2).

These cities are classified as various types in terms of their principal urban function, including waterway ports, railway ports, and highway ports, among which there are 22 first- and second-level ports to Russia that are cleared by customs services (Table 1). In 2019, these border cities covered an area of 590,800 km^2^, with a total population of 16.65 million, and the GDP was 588.54 billion RMB, accounting for 47.20%, 14.43%, and 10.6% of the total of these factors for Northeast China, respectively. On the Russian side, there are five border regions from south to north: Primorye Territory, Khabarovsk Territory, Jewish Autonomous Region, Amur Region and Trans-Baikal Territory. In 2019, the border regions were 1,785,400 km^2^, the population was 5.22 million people, and the GDP was 237.84 billion RMB, accounting for 25.68%, 63.89%, and 42.82% of the total of these factors for Far East Russia, respectively [55]. The bilateral trade between China and Russia exceeded 948.66 billion RMB in 2021, and this went up by 26.6% each consecutive year. Bulk commodities are mainly traded through the border ports which show huge potential.

In order to leverage the comparative advantages of the border regions between China and Russia, the two governments signed “the Plan for China-Russia Cooperation and Development in the Russian Far East (2018–2024)” in 2018. In the border regions, the construction of the Heihe-Blagoveshchansk road bridge, the Tongjiang-Nizhni Leninskoye railway bridge, and other international transport corridors have been accelerated. “The Border Development and Opening Plan of Heilongjiang and Northeastern Inner Mongolia” which was issued by the Chinese government in 2013 was highly compatible with “the Social and Economic Development Strategy of the Russian Far East and Baikal Region in 2025” that was proposed by the Far East Russia in 2009, indicating that the border regions have become key areas for bilateral cooperation. However, the outbreak of COVID-19 has brought new opportunities and challenges to the geo-environment, so the measurement of its economic resilience has become a new direction to study the sustainable development of the border regions.

### 3.2. Methodology and Data

#### 3.2.1. Methodology

(1) Economic Resilience Measurement

This study draws on the widely adopted regional economic resilience measurement method that was proposed by Martin and Gardiner [56]. Most of the studies adopt the resistance and recovery dimensions of resilience for empirical research, thus dividing the development stages of the regional economy into a recession period and recovery period. In this paper, because the border area was still in the early stage of the pandemic, its resistance to the recession was mainly measured. The calculation formula is as follows:

(1) $$R_{i}^{t}=\left(\Delta Y_{i}-\Delta E\right)/\left|\Delta E\right|$$

where $R_{i}^{t}$ is the relative resilience level of the *i*th research object in the *t* year; $\Delta Y_{i}$ is the actual economic situation of the *i*th research object, as shown in Formula (2); ΔE is the predicted economic situation of the research object based on the economic condition of the whole region, as shown in Formula (3).

(2) $$\Delta Y_{i}=Y_{i}^{t}-Y_{i}^{t-k}$$

(3) $$\Delta E=((Y_{r}^{t}-Y_{r}^{t-k})/Y_{r}^{t-k})Y_{i}^{t-k}$$

where $Y_{i}^{t}$ and $Y_{i}^{t-k}$ are the quantitative indexes of the research object *i* (city or economic region) at t and t−k year, respectively. $Y_{r}^{t}$ and $Y_{r}^{t-k}$ are the quantitative indexes of the region (economic region or country) where the research object is located at t and t−k year, respectively. Then, the resilience can be expressed as follows:

(4) $$R_{i}^{t}=\frac{(Y_{i}^{t}-Y_{i}^{t-k})/Y_{i}^{t-k}-(Y_{r}^{t}-Y_{r}^{t-k})/Y_{r}^{t-k}}{\left|(Y_{r}^{t}-Y_{r}^{t-k})/Y_{r}^{t-k}\right|}$$

where $R_{i}$ refers to the relative economic resilience of each research object. When $R_{i}$ > 0, the economic resilience of the object i exceeds the average level, and the larger the value is, then the better the performance it has in the research object, and vice versa.

(2) Geographical Detector Model

The geographical detector model is a tool that is used to detect the spatial differentiation and driving force of geographical phenomena [57]. The factor detection module and interactive detection module are applicable to identify the explanation power of different socio-economic factors for the explained variables. The calculation model is as follows:

(5) $$R=1-\frac{1}{N\sigma^{2}}\overset{L}{\underset{h=1}{\sum }}N_{h}\sigma_{h}^{2}$$

where q is the determinant index of the changing pattern of economic resilience, with the value range of [0,1]. h = 1, 2, …, *L* are the classification numbers of the influencing factors, $N_{h}$ and N are the units numbers of the h-type city and the total number of cities in the whole region, respectively, $\sigma_{h}^{2}$ and $\sigma^{2}$ are the variances of the h-type cities and the whole region, respectively. The larger the q value, then the higher the explanatory degree of the factors for resilience is.

The previous findings have shown that the resilience of the urban economic systems is of great relevance for many mechanisms [42,46,58]. Based on recent studies, combined with the inherent nature of the border areas and the impacts of the COVID-19 pandemic, this paper selected 10 indicators (Table 2) from five spheres, development foundation, economic structure, financial environment, innovation ability, and policy support, to explore how these variables shaped the response to the changes in the economic resilience of the study area from 2019 to 2020.

#### 3.2.2. Data Sources

In order to compare the variation that occurred before and after the COVID-19 pandemic, our research period was 2019–2020, and the research area was of 10 prefecture-level border cities (autonomous prefecture, league) in Northeast China. The vector data of the administrative area came from the standard map service website of The State Bureau of Surveying and Mapping (http://bzdt.ch.mnr.gov.cn/, accessed on 10 November 2021) [59]. The socio-economic data were derived from the statistical yearbook of each province and prefecture-level city and the statistical bulletins of the national economy and social development from 2019 to 2021. The statistical data on the border regions of the Far East Russia were derived from the Russian Federal State Statistics Service, Rosstat (http://bzdt.ch.mnr.gov.cn/, accessed on 10 November 2021) [60], and the RUSSIAN STATISTICAL YEARBOOK Russian Statistical Yearbook 2020.4. 

## 4. Results

### 4.1. Diffe rentiated Economic Resilience under COVID-19

#### 4.1.1. Economic Variation in the Border Region

**Figure 3:**
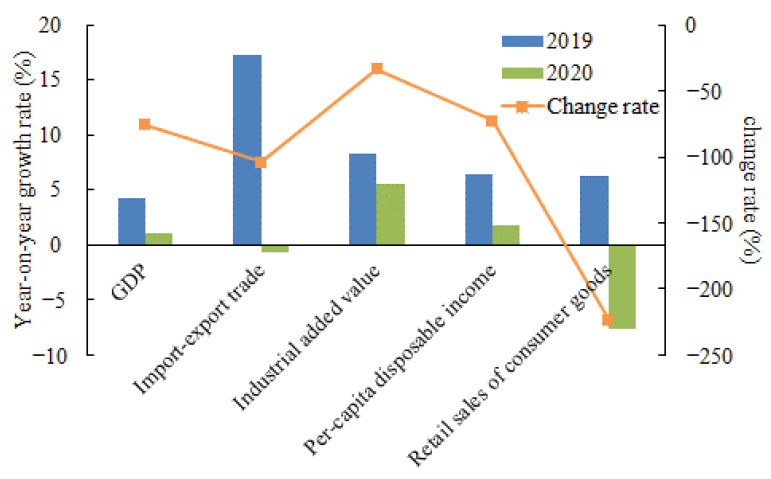
Variation in the growth rates of major economic indicators in 2019–2020.

Under the impact of the pandemic, the main economic indicators of the Sino-Russian border area changed significantly (Figure 3). External shocks inevitably led to a significant decline in the growth rates of all of the economic indicators, including the GDP, the total import–export trade value, the industrial added value above scale, the per-capita disposable income of the urban residents, and the total retail sales of consumer goods.

On the whole, the economic crisis that was caused by COVID-19 led to a sharp drop in all of the supply-side and demand-side indicators. The GDP growth rate, which represents the whole economic situation of the region, dropped from 4.24 to 1.04% under the impact of the pandemic, which represents a decline of 75.47%. The total import–export trade, as a direct manifestation of the export-oriented economy, bore the brunt of the epidemic due to the border blockade, and the growth rate changed from positive to negative, with a decrease of −103.83%. The growth rates of the industrial added value above scale and the per-capita disposable income of the urban residents fell by 33.25 and 72.65%, respectively. The total retail sales of the consumer goods were most severely affected by the pandemic, with the growth rate dropping from 6.24 to −7.68%, which represents a decline of 223.08%. The average growth rates of the five economic indicators decreased significantly from 2019 to 2020, and this totaled 101.66%. At the same time, the variation of each indicator exhibited a differentiation in the regional economic resilience. In 2019, all of the economic indicators of the studied area experienced positive growth, but by 2020, the growth rates of most of the indicators approached the threshold of a zero value and even showed a negative rebound. Among them, the total import–export trade value and the total retail sales of the consumer goods were the most affected by the domestic and foreign financial markets, so they were the first variables to show vulnerability and sensitivity that resulted in a significant decline. Comparatively speaking, under efficient governance to support key industrial enterprises, the industrial added value above scale gradually recovered, which caused the growth rate to show a slight downward trend. The ranges of the GDP growth rate and the per-capita disposable income growth rate of the urban residents are obvious and show a trend of convergence.

#### 4.1.2. GDP Resilience

**Figure 4:**
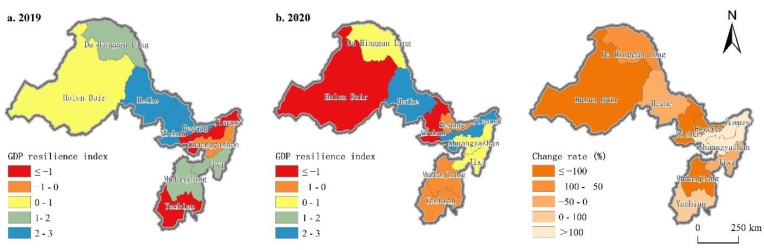
Differentiation of GDP resilience in China–Russia border cities from 2019 to 2020.

Figure 4 shows that there is significant spatial differentiation in terms of GDP resilience values. In 2019, the region’s GDP resilience was high. The number of cities with positive resilience accounted for 70%, and their average value and standard deviation were 0.89 and 2.37, respectively. Among them, the GDP resilience values of Hegang, Heihe, and Yichun were all greater than two, which were higher than those of other border cities, showing significant spatial agglomeration, while the GDP resilience indexes of Jiamusi, Shuangyashan, and Yanbian were negative, with Jiamusi performing the worst among them (−3.28). In 2020, the area showed significant differences in terms of the economic resilience against the pandemic. The coefficient of variation of the GDP resilience increased dramatically from 2.66 to 12.10, this indicating that there was a significant divergence. The proportion of the cities with a positive index decreased by 28.57%, and the average value and standard deviation decreased sharply, and these were 0.11 and 1.36, respectively. Heihe and Jiamusi achieved a positive growth in the fixed asset investment, and this promoted the transformation and upgrading of the leading industries in terms of their equipment and building materials through financial subsidies, so they showed strong economic resilience. Whereas the index for Da Hinggan Ling, Shuangyashan, and Jixi approached a negative value. Yanbian, Jiamusi, and Shuangyashan through a series of tax reduction policies, promoted the resumption of their production and their market, thus showing a significant increase in their economic resilience. Meanwhile, the resilience indexes of Hulun Buir and Yichun decreased the most, which were already at a low level to begin with. Overall, due to the impact of the COVID-19 pandemic, the number of regions with a reduced GDP resilience index accounted for 70%. Among them, the secondary and tertiary industries in Hulun Buir, Yichun, Hegang, and Mudanjiang had significant negative effects, and the GDP resilience indexes declined the most, all of which exceeded 100%.

#### 4.1.3. Multi-Dimensional Economic Resilience

**Figure 5:**
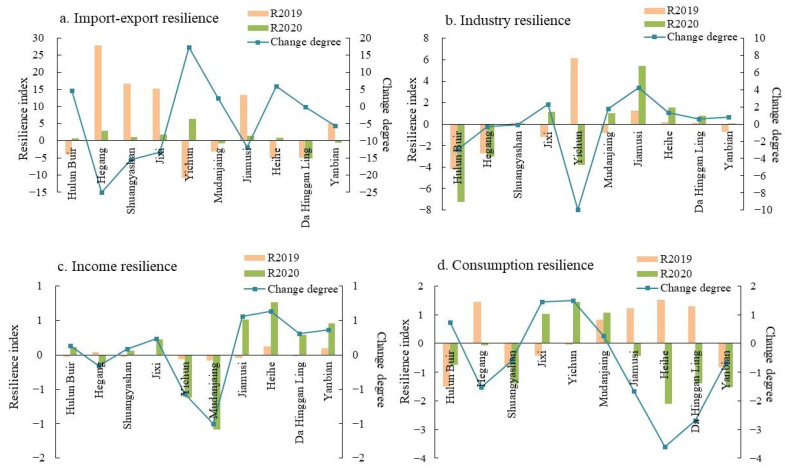
Import–export resilience, industrial resilience, income resilience and consumption resilience.

This study further compares the relative resilience of the studied area from four dimensions: import and export trade, industry, income, and consumption (Figure 5). Overall, the import and export resilience declined the most, while the income resilience was relatively stable. However, there was significant heterogeneity in the different dimensions among the different regions.

From the perspective of import and export resilience (Figure 5a), the whole resilience level of the border area in 2019 was relatively high, with an average value of 5.04. Hegang, Shuangyashan, and Jixi showed a higher import and export resilience, all of which exceeded 15; however, Yichun was the weakest one. In 2020, the import and export resilience of the border area decreased drastically to 0.91, while Yichun ranked the highest due to the robust import of iron ore powder. However, while it was affected by the port closure and suspension of timber logging, the export trade decreased by 29.1% year-on-year, with there being a significant trade deficit. The resilience indexes of most of the other cities were positive, but the gap between the cities was narrower than it was in 2019. As it was affected by the fluctuations in the commodity prices, the import and export resilience levels in Da Hinggan Ling, Mudanjiang, and Yanbian were lower than the regional average was, among which the resilience index in Da Hinggan Ling was the lowest, with it reaching −5.23.

In terms of the industrial resilience (Figure 5b), Yichun was in the national key ecological function zone and was in a period of economic transformation, so its ability to resist the crisis was relatively weak, with values that decreased from 6.14 to −3.83 and a decline rate of 162.40%. In this city, the added value of the mining industry decreased the most, with it reaching 41.20%. The economic structure of Hulun Buir and Hegang, which were dominated by heavy industry, led to a higher output loss due to the rising prices of the raw materials, thereby showing a lack of resistance. The governments of Jixi, Jiamusi, Heihe, and Mudanjiang implemented a series of tax and fee reduction measures for the industrial enterprises, which acted as a series of “shock absorber” to withstand the crisis. Among them, Jixi and Mudanjiang increased their financial funds for the industrial enterprises to resume work and production, and continued to relax the market accessibility, making the industrial resilience indexes turn from negative to positive.

The income resilience values were lower than the other indicators were (Figure 5c). However, with the subsidy of a series of special government funds, the employment situation gradually stabilized, thereby making the income resilience in most cities perform better than they would have done. In 2019, the income resilience index had little spatial difference and remained at around −0.1–0.1. However, in 2020, the income resilience level of Mudanjiang and Yichun decreased, while that of Jiamusi, Heihe, and Yanbian increased slightly with the support of various livelihood guarantee policies.

In terms of consumption resilience (Figure 5d), the indexes of Hegang, Jiamusi, Heihe, and Da Hinggan Ling turned from positive to negative, among which Heihe was the most severely impacted (−2.10 in 2020). This is because the consumption and retail markets in Heihe mainly rely on the residents’ consumption, foreign trade, and tourism, which were confronted with a severe downturn in the early stage of the pandemic, and since this, the consumer market’s recovery has been uncertain. The consumption resilience in Hulun Buir and Shuangyashan has remained low because the local population scale and structure have hindered the development of the consumer market, and the contribution of traditional consumer goods has been weakened. The values of Jixi and Yichun turned from negative to positive, and the consumer markets there demonstrated a stronger recoverability.

#### 4.1.4. Classification of Border Regions by Shocks

**Figure 6:**
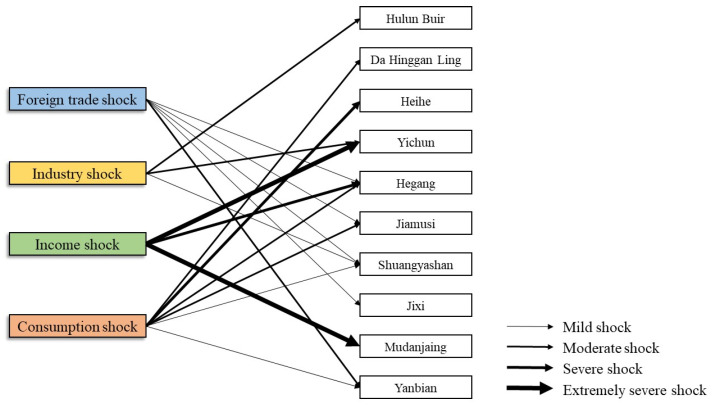
Classification of shock types.

Cities with a decline rate of more than 50% in certain resilience indexes can be considered as being significantly affected by this type of shock. Accordingly, the 10 cities are divided according to the foreign trade shock, the industry shock, the income shock, and the consumption shock (Figure 6). Under each shock type, the severity of the damage was reflected in the variation range of the resilience indexes. Using the Arcgis10.2 (Established by Jack Dangermond and Laura Dangermond, Redlands, California, CA, USA )natural discontinuity classification method, the area was further divided into four categories under each dimension: mild shock (−50–−100%), moderate shock (−100–−250%), severe shock (−250–−400%), and extremely severe shock (<−400%). On the basis of the main types of shock and degrees of influence that they had, we can identify the potential classification of the border area as it was impacted by the COVID-19 pandemic.

As shown in Figure 6, the number of cities that were affected by the foreign trade shock accounts for the largest proportion, including Hegang, Jiamusi, Shuangyashan, Jixi, and Yanbian. Among them, Yanbian suffered the most, as its economy is highly dependent on trade with and investment from the foreign economy, so the severe foreign trade restriction meant that the import–export value of Yanbian was greatly reduced. The industry shock cities include Hulun Buir, Yichun, and Shuangyashan. For Hulun Buir and Yichun, their industry excessively relies on resource processing and manufacturing, with a low-added value and a poor sustainability forming a moderate shock. Evidence has shown that the number of cities with an income shock is relatively small, but in terms of the degree of this shock, Yichun, Hegang, and Mudanjiang exhibited a higher income vulnerability, among which Hegang experienced a severe shock, while Yichun and Mudanjiang experienced an extreme shock. In regard to the consumption shock, 60% of the border cities were affected by the consumption shock, but the shock was mainly mild and moderate, while Heihe received a severe consumption hit under the influence of the accommodation and catering sectors.

Overall, the cities with a foreign trade shock and a consumption shock account for the largest proportion, indicating that in the face of the pandemic, import–export trade and the retail of consumer goods were the most vulnerable economic sectors in the borderland. The number of cities that were affected by an industrial shock or an income shock is relatively small, but the cities with an income shock show a weaker resistance and dynamism.

### 4.2. Heterogeneity of Economic Resilience against COVID-19: What Matters?

#### 4.2.1. Driving Factors of Resilience in the Whole Area

To capture the determinants of change in economic resilience from 2019 to 2020, the border cities with a reduced GDP resilience were selected as samples. We took the change rate of resilience as the dependent variable, and then we classified the independent variables by using the ArcGIS10.2 Jenks method. The Pearson correlation coefficient was measured to reveal the action direction between the dependent and independent variables. The degree of influence of each factor is shown in Table 3.

Table 3 shows that the economic openness (0.774), the fiscal expenditure (−0.718), and the asset investment (−0.637) all have a higher explanatory power, and all of them pass the significance test. There is a positive correlation between the economic openness and the reduction in the GDP resilience; that is, the higher the economic openness is, then the stronger the negative impact on the regional economy is. This is consistent with the relevant research findings that a more extensive global connectivity in the supply demand chain presents a higher vulnerability during an economic crisis [46,61,62]. Meanwhile, as the embodiment of the government measures, the asset investment and the fiscal expenditure are important drivers in promoting regional trade and investment activities, so they have a strong explanatory power for maintaining economic resilience. The regional economic resilience to the COVID-19 pandemic greatly tested the governance capacities and coping strategies [63,64]. The central and local governments implemented a series of funds projects, such as the anti-epidemic national debt, thereby actively exerting the leverage effect of the funds on the industrial chains. Geographical location (0.622), industrial advancement (−0.528), and research input (−0.578) were also important driving forces for the change that occurred in regional economic resilience. Provincial capital cities are conducive to realizing the joint development between the border and the inland resources, industry, market, logistics, information, and other spheres. Therefore, the farther the distance between the border regions and the provincial capitals is, then the greater the extent to which the borderland economy will be shocked. The degree of industrial advancement and research input, as the embodiment of transformation and innovation ability in regional economies, are negatively correlated with the vulnerability and sensitivity of the border area to an economic crisis. At the same time, it should also be noted that industrial diversity has a positive effect on the reduction in the regional economic resilience. Relevant studies [65] have found that the industrial structures with a higher diversification are more vulnerable at the early stage of a crisis. Therefore, it can be considered that areas with a higher degree of overall industrial diversity may suffer from more severe short-term economic shocks. In addition, it is surprising that the effects of the urbanization rate, market potential, and education input on GDP resilience are not significant.

**Figure 7:**
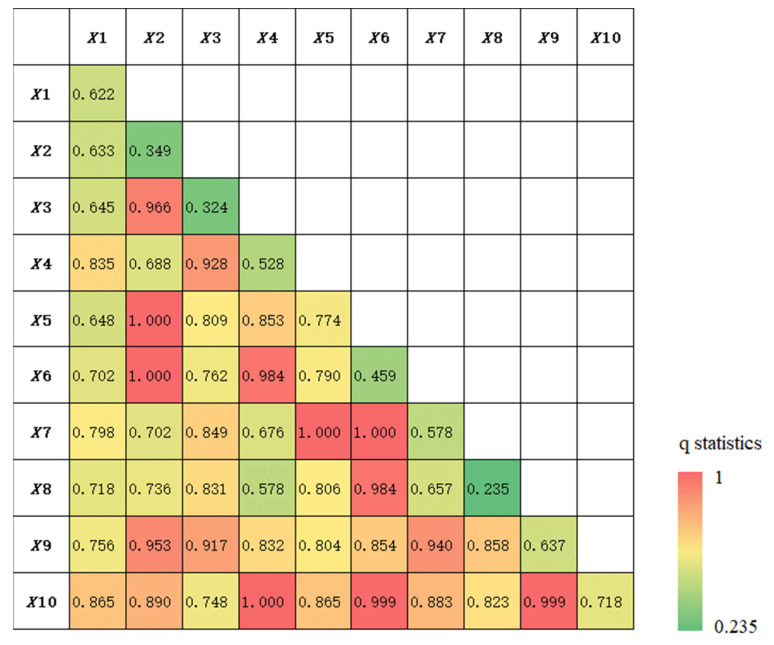
Interaction results of influencing factors.

Figure 7 shows the q statistics of the interaction detector. Various elements interact, flow, and correlate with each other in the border area. The interaction result between any of the two impact factors showed a nonlinear and bi-factor enhancement, and this was significantly stronger than that of a single individual factor. On the whole, asset investment and fiscal expenditure in policy support factors are the main driving forces supporting the regions against economic shocks, and the coupling interactions with the other factors have a synergistic enhancement effect. At the same time, economic openness and market potential in the financial factors, as special interference factors that reflect the region’s resistance to the COVID-19 pandemic, are the most tightly interwoven factors, but they also have a synergistic enhancement effect on regional economic resilience.

#### 4.2.2. How Factors Matter for Economic Resilience and Different Types of Economic Shock

Combined with the classification of border regions in Section 4.1.4, this paper further analyzed the action of each influencing factor on the different types of economic shock. The results of this are shown in Table 4.

For regions that were exposed to the foreign trade shock, market potential, economic openness, and industrial advancement were the key driving factors that significantly affected the change in import–export resilience. As the gateway for foreign trade, logistics, and capital information, the border regions were most vulnerable to the impact of economic openness and the market environment in the context of the border blockades and trade control that resulted from the COVID-19 pandemic, and so both of these impacts showed positive driving effects. At the same time, the proportion of the secondary and tertiary industries in cities that were exposed to the foreign trade shock was relatively large. This kind of foreign trade mostly shows the non-relevant diversity of the industries. Due to almost all of the import and export activities being blocked during epidemic, the border cities exhibited a significant decline in their foreign trade resilience, which was not conducive to withstand the crisis. In the structural adjustment period, the regions with more advanced industrial structures quickly shifted their development paths and fostered stronger resilience. Therefore, industrial advancement negatively drives the decline of import–export resilience.

The border cities with the industry shock were mainly resource-based regions, and their industrial structures were mainly diversified. In other words, these were different industries that were still closely related in terms of their products and technologies. At the same time, the government agencies adopted a series of financial subsidies and measures for the industry, which was conducive to the resource-based cities to be able to resist the industry shock. Therefore, industrial diversity is a negative factor that significantly impacted the change in the industry resilience. In addition, the market potential and the fiscal expenditure are indeed powerful forces that significantly affected the industrial resilience. As an important part of the old industrial base in Northeast China, the border area is supported by a series of national financial measures, which act as risk absorbers to improve the industry resilience.

The quantitative evidence implies that there is a set of factors that are key to achieving income resilience. Provincial capital cities have a significant impact on the improvement of the border residents’ incomes. Therefore, the distance between the border regions and the provincial capital cities had a positive synergistic effect on the change in the income resilience. In addition, the domestic and foreign financial environment also matter to the local income, so ensuring market stability and improving the market environment is of great significance to promoting local employment and income. Finally, the regional consumption capacity can be regarded as the response of the per-capita disposable income level. The factors impacting the consumption of the cities are consistent with those impacting the income of cities, but the force of each variable is relatively weak.

Conceptually, the border region is a human-land coupling system with openness, complexity, and market potential factors had a profound impact on its economic resilience. At the same time, as an important part of the old industrial base in Northeast China, the border area also has internal contradictions, such as a rigid administrative system and single economic structure, which lead to a lack of economic vitality and a depressed consumer market, thus it became a bottleneck of restricting the development of the local industry, income, and consumption resilience. The coordinated development of the provincial capital cities and the border areas can allow for a integration in key fields such as industrial enterprise, facility connectivity, and energy security, and it can facilitate a positive interaction between the border trade and hinterland advantages. Moreover, due to the limitations that are associated with its environmental conditions, geographical location, development history, and economic development level, the population density of the Sino-Russian border area is gradually decreasing, and the outgoing population is composed of mostly young adults. Although this alleviates the direct impact that is caused by the short-term economic shocks to some extent, it creates long-term obstacles in the development of economic resilience and the security of the national frontier.

## 5. Conclusions and Discussion

Regional economic resilience is a multi-actor, contextualized, and non-equilibrium process [16]. The key research theme of it is understanding why some regions are more capable of withstanding shocks than others are, and what determinants shape the response to uncertainty [66]. Under the impact of the COVID-19 pandemic, regional economic resilience has attracted more and more attention from academic circles. The economic development of the border regions between China and Russia has been one of the major strategies for the current national economic development. Therefore, it is particularly important to accurately assess the economic resilience of the border regions. However, most studies on regional economic resilience and its influencing factors have focused on the 2008 international financial crisis, while few have focused on the impact of the COVID-19 pandemic. At the same time, the current measurement dimension of regional economic resilience has been relatively single, and the analytical perspective has focused too much on the internal region, while it has ignored the impact of global-local linkage. Therefore, in this paper, we took the China–Russia border regions, which are both vulnerable and sensitive to shocks, to be the research object. By constructing a theoretical framework of regional economic resilience, this paper explored the response characteristics of GDP resilience, import–export resilience, industry resilience, income resilience, and consumption resilience in the face of the COVID-19 pandemic and identified the determinants of economic resilience in the cities that experienced different types of shocks and their direction of action.

According to the results of our study, the following conclusions can be drawn: (1) The economic crisis that was caused by the COVID-19 pandemic had wide-ranging economic impacts in the border region. The import–export trade and retail sales of consumer goods were the first indicators to show the vulnerability and sensitivity to the pandemic. The GDP growth rate, the industrial added value above scale, and the per-capita disposable income of the urban residents also presented a downward trend. Overall, the entire borderland economy is in the downward stage of the resistance period. (2) In terms of the multi-dimensional resilience, the import and export resilience declined the most, while the industrial resilience and income resilience of most of the cities increased against the trend, thus showing that they had good resistance against the COVID-19 shock. (3) Borderland economic resilience is a spatially heterogeneous phenomenon, and the economic resilience of each border city exhibited differentiation during the epidemic. This suggests that the geographies of the economic resilience in different dimensions are complex and contextualized. As such, it is useful from a policy perspective to classify them by the main types of shocks (foreign trade, income, industry, and consumption). (4) There are multiple factors shaping borderland resilience. Economic openness, fiscal expenditure, and asset investment matter the most. Meanwhile, the interaction between the impact factors presents nonlinear and bi-factor enhancement effects. The drivers’ effects on various shock types are uneven, but the degree of the impact of economic openness and market potential factors is always profound. By exploring the characteristics and influencing factors of economic resilience amidst the COVID-19 pandemic in the China–Russia border regions, this paper provides a new analytical perspective for the resilience research, and also helps local governments to better formulate relevant economic policies. The economic crisis that was caused by the COVID-19 pandemic has led to a sharp decline in the resilience indicators of the supply and demand sides, but different types of shocks have significantly different ways and degrees of impacting the regional economy. China’s border cities are highly dependent on foreign trade, so import and export trade were affected first when the COVID-19 outbreak occurred. The impact that was caused by the COVID-19 pandemic affected almost all of the foreign trade industries in the short term. Therefore, the diversified industrial structure and high degree of openness of foreign trade did not play the roles of “shock absorber”, but they had a negative impact. At the same time, the China–Russia border regions mostly belong to the resources cities, wherein the proportion of the secondary industries is too high. The relatively large state-owned economy makes it more protected by government policies when the area is dealing with a crisis, which is conducive to the recovery and development of the industry. Therefore, in the short term, the diversified industrial structure is not conducive to the regional resistance to shocks, while the specialized economic structure has the opposite effect under the role of government agents, which has a significant promoting effect on the improvement of regional economic resilience.

Peace, stability, and socio-economic development in the border areas are of national interest to China. The COVID-19 pandemic has had a great impact on the process of globalization. Although the pandemic has been effectively contained in China at present, exogenous and local risks still exist. However, the regional economy during the COVID-19 pandemic was not simply about the negative impact, but rather it offered agency to try out renewed growth paths [1,16]. As it was mentioned earlier, regional economic resilience is very much up to positive intervention policies. This requires the government and agencies to comprehensively consider the uncertain impact that border regions may face in the future, adopt corresponding industrial development policies, and promote the coordinated development of diversification and specialization through the rational allocation of the industrial structure. Therefore, in the process of the high-quality development of the border regions, we should not only pay attention to the cohesion and cooperation between the industries, but also ensure the spillover and dispersion of the shocks. At the same time, government departments should focus on manufacturing in the border regions to improve the technological content and market competitiveness of the export industries. Moreover, policymakers need to ensure the specialized transformation of the border ports and develop the comparative advantages of warehousing, logistics, and service trade, thereby forming a composite ports economic system. Taking a place-specific and time-specific view of the events that occurred during the COVID-19 pandemic, multi-actors should not merely encourage the export-oriented enterprises to turn to the domestic market but also pursue the digital transformation of it and promote cross-border e-commerce trade with Russia, thereby building resistance to the crises that stem from international economic uncertainties that were caused by the pandemic.

Our research also has deficiencies. Due to the limitation of the research period, our study on the regional resilience during the COVID-19 pandemic is mainly reflected by the short-term dimension of resistance and recovery. The resistance and recovery of the regional economic systems that faced varied shocks can be understood by quantitative measures, but their reorganization and renewal after a disturbance often take a long time to evaluate. When analyzing the current global disasters such as the COVID-19 pandemic, it should be noted that this global crisis often includes several shocks, such as a rapid economic recession which is caused by short-term liquidity reduction and further shrinkage which is caused by a decline in the long-term market demand. Therefore, when examining the impact of specific crises in the future, these shocks should be studied as a whole [67].

## Figures and Tables

**Table 1:** The types of border ports to Russia.

Province	Prefecture-Level City	Port Name	Type
Inner Mongolia	Hulun Buir	Manzhouli	Railway, Highway
		Heishantou	Highway
		Shiwei	Highway
Heilongjiang	Hegang	Luobei	Waterway
	Shuangyashan	Raohe	Waterway
	Jixi	Hulin	Highway
		Mishan	Highway
	Yichun	Jiayin	Waterway
	Mudanjiang	Suifenhe	Railway, Highway
		Dongning	Highway
	Jiamusi	Fujin	Waterway
		Tongjiang	Waterway
		Fuyuan	Waterway
	Heihe	Heihe	Waterway
		Sunwu	Waterway
		Xunke	Waterway
	Da Hinggan Ling	Mohe	Waterway
		Huma	Waterway
Jilin	Yanbian	Hunchun	Railway, Highway

**Table 2:** Variables and descriptive explanation.

Classification	Variable	Definition	Unit
Development Foundation	Geographical Location	Distance from provincial capital city	km
	Urbanization Rate	Urban population/Total population	%
Economic Structure	Industrial Diversity	Herfindahl-Hirschman index	/
	Industrial Advanced	Second and third industries added value/GDP	%
Financial Environment	Economic Openness	Actual utilization of foreign capital/GDP	%
	Market Potential	Population density	person/km^2^
Innovation Capability	Research Input	Science-technology expenditure/Public financial expenditure	%
	Education Input	Education expenditure/Public financial expenditure	%
Policy Support	Assets Investment	Per capita fixed assets investment	104 CNY
	Fiscal Expenditure	Per capita local fiscal expenditure	104 CNY

**Table 3:** Determinants of economic resilience change.

Variable	q Statistic	*p*-Value
Geographical Location (*X*_1_)	0.622 (+)	0.056
Urbanization rate (*X*_2_)	0.349 (−)	0.549
Industrial Diversity (*X*_3_)	0.324 (+)	0.570
Industrial Advancement (*X*_4_)	0.528 (−)	0.123
Economic Openness (*X*_5_)	0.774 (+) **	0.004
Market Potential (*X*_6_)	0.459 (+)	0.230
Research Input (*X*_7_)	0.578 (−)	0.144
Education Input (*X*_8_)	0.235 (−)	0.629
Assets Investment (*X*_9_)	0.637 (−) *	0.048
Fiscal Expenditure (*X*_10_)	0.718 (−) *	0.033

Note: * means significance below 0.05, ** means significance below 0.01. "+" means that the factor plays a positive role, and "−" means that the factor plays a negative role.

**Table 4:** Determinants of economic resilience change pattern in different types.

	Foreign Trade Shock	Industry Shock	Income Shock	Consumption Shock
Geographical location (*X*_1_)	0.532 (−)	0.669 (+)	0.981 ** (+)	0.633 * (+)
Urbanization rate (*X*_2_)	0.387 (−)	0.626 (−)	0.144 (−)	0.246 (−)
Industrial diversity (*X*_3_)	0.737 (+)	0.981 ** (−)	0.977 ** (−)	0.685 * (−)
Industrial advancement (*X*_4_)	0.926 * (−)	0.855 (−)	0.635 (+)	0.467 (+)
Economic openness (*X*_5_)	0.975 ** (+)	0.586 (+)	0.976 * (+)	0.962 ** (+)
Market potential (*X*_6_)	0.980 ** (+)	0.992 * (+)	0.948 * (+)	0.729 * (+)
Research input (*X*_7_)	0.748 (−)	0.578 (−)	0.812 (−)	0.505 (−)
Education input (*X*_8_)	0.020 (−)	0.440 (−)	0.271 (−)	0.410 (+)
Assets investment (*X*_9_)	0.555 (−)	0.104 (−)	0.902 * (−)	0.259 (−)
Fiscal expenditure (*X*_10_)	0.949 (−)	0.991 * (−)	0.366 (−)	0.497 (−)

Note: * means significance below 0.05, ** means significance below 0.01. "+" means that the factor plays a positive role, and "−" means that the factor plays a negative role.

## Data Availability

The data used to support the findings of this study are available from the corresponding author upon reasonable request.

## References

[1] Nicola M., Alsafi Z., Sohrabi C., Kerwan A., AI-Jabir A., Iosifidis C., Agha M., Agha R. (2020). The socio-economic implications of the coronavirus pandemic (COVID-19): A review. Int. J. Surg..

[2] Vidya C.T., Prabheesh K.P. (2020). Implications of COVID-19 pandemic on the global trade networks. Emerg. Mark. Financ. Trade.

[3] Barlow P., van Schalkwyk M.C., McKee M., Labonté R., Stuckler D. (2021). COVID-19 and the collapse of global trade: Building an effective public health response. Lancet Planet. Health.

[4] Harrison J., Delgado M., Derudder B., Anguelovski I., Montero S., Bailey D., de Propris L. (2020). Pushing regional studies beyond its borders. Reg. Stud..

[5] Prokkola E.K. (2019). Border-regional resilience in EU internal and external border areas in Finland. Eur. Plan. Stud..

[6] Li H., Lo K., Zhang P. (2020). Population shrinkage in resource-dependent cities in China: Processes, patterns and drivers. Chin. Geogr. Sci..

[7] Hossain M.P., Junus A., Zhu X., Jia P., Wen T.H., Pfeiffer D., Yuan H.Y. (2020). The effects of border control and quarantine measures on the spread of COVID-19. Epidemics.

[8] Radil S.M., Castan Pinos J., Ptak T. (2021). Borders resurgent: Towards a post-Covid-19 global border regime?. Space Polity.

[9] Kajta J., Opiłowska E. (2021). The Impact of Covid-19 on Structure and Agency in a Borderland. The Case of Two Twin Towns in Central Europe. J. Borderl. Stud..

[10] Martin R., Sunley P. (2015). On the notion of regional economic resilience: Conceptualization and explanation. J. Econ. Geogr..

[11] Giannakis E., Bruggeman A. (2020). Regional disparities in economic resilience in the European Union across the urban–rural divide. Reg. Stud..

[12] Hassink R. (2010). Regional resilience: A promising concept to explain differences in regional economic adaptability?. Camb. J. Reg. Econ. Soc..

[13] Hu X.H., Hassink R. (2020). Adaptation, adaptability and regional economic resilience: A conceptual framework. Handbook on Regional Economic Resilience.

[14] Martin R., Sunley P., Bristow G., Healy A. (2020). Regional economic resilience: Evolution and evaluation. Handbook on Regional Economic Resilience.

[15] Tan J.T., Hu X.H., Hassink R., Ni J. (2020). Industrial structure or agency: What affects regional economic resilience? Evidence from resource-based cities in China. Cities.

[16] Hu X.H., Li L.G., Dong K. (2022). What matters for regional economic resilience amid COVID-19? Evidence from cities in Northeast China. Cities.

[17] Golunov S., Smirnova V. (2022). Russian border controls in times of the COVID-19 pandemic: Social, political, and economic implications. Probl. Post-Communism.

[18] Sun J.W. (2020). The impact of the new crown pneumonia epidemic on China’s regional economic development. Reg. Econ. Rev..

[19] Shao S. (2020). Analysis of China’s novel coronavirus pneumonia epidemic based on previous PHEIC events. Int. Invent. Sci. J..

[20] Wen Y., Zhang T., Du Q.Y. (2020). Quantifying the Covid-19 economic impact. SSRN Electron. J..

[21] Song T. (2018). Research framework of border geography from the perspective of geo-economics. Sci. Technol. Her..

[22] Song T., Liu W.D., Li L. (2016). International research on the border regions with a geopolitical perspective and revelation. Prog. Geogr..

[23] Song T., Cheng Y., Liu W.D., Liu H. (2017). The spatial disparity and impact mechanism of geo-economy in the border areas of China. Acta Geogr. Sin..

[24] Kolosov V., Sebentsov A. (2020). Russian borderlands: Contemporary problems and challenges. Reg. Sci. Policy Pract..

[25] Song Z.Y., Zhu Q.L. (2020). Spatio-temporal pattern and driving forces of urbanization in China’s border areas. Acta Geogr. Sin..

[26] Zhu Y.Y., Wang S.J., Feng Z.X. (2011). The central place system structure and formation mechanism research of the Chinese Northeastern border area center. Econ. Geogr..

[27] Cong Z., Yu T. (2010). The investigation of the frontier ports economy of the East of Northeast. Econ. Geogr..

[28] Zhou D.J., Xu S. (2021). The Spatial Development Path of China’s Border Areas in the 14th Five-Year Plan Period. Urban Dev. Stud..

[29] Martin R. (2012). Regional economic resilience, hysteresis and recessionary shocks. J. Econ. Geogr..

[30] Grabner S.M., Wink R. (2021). Regional economic resilience: Review and outlook. Economic Resilience in Regions and Organisations.

[31] Robert H., Gong H.W., Kobayashi A. (2020). Regional resilience. International Encyclopedia of Human Geography.

[32] Hu X.H., Hassink R. (2017). Exploring adaptation and adaptability in uneven economic resilience: A tale of two Chinese mining regions. Camb. J. Reg. Econ. Soc..

[33] Chen M.Y. (2017). An international literature review of regional economic resilience: Theories and practices based on the evolutionary perspective. Prog. Geogr..

[34] Pike A., Dawley S., Tomaney J. (2010). Resilience, adaptation and adaptability. Camb. J. Reg. Econ. Soc..

[35] Joachim M. (2019). Governance for the Sustainable Development Goals: Exploring an Integrative Framework of Theories, Tools and Competencies.

[36] Li Y., Chen W., Sun Y. (2019). New thinking on regional resilience analysis of geography from the perspective of correlation evolution. Geogr. Res..

[37] Martin R.L. (2018). Shocking aspects of regional development: Towards an economic geography of resilience. The New Oxford Handbook of Economic Geography.

[38] Boschma R. (2015). Towards an evolutionary perspective on regional resilience. Reg. Stud..

[39] Simmie J., Martin R. (2010). The economic resilience of regions: Towards an evolutionary approach. Camb. J. Reg. Econ. Soc..

[40] Li L.G., Zhang P.Y., Li X. (2019). Regional economic resilience of the old industrial bases in China—A case study of Liaoning Province. Sustainability.

[41] Klimanov V.V., Kazakova S.M., Mikhaylova A.A. (2020). Economic and fiscal resilience of Russia’s regions. Reg. Sci. Policy Pract..

[42] Tan J.T., Zhao H.B., Liu W.X., Zhang P.Y., Qiu F.D. (2020). Regional economic resilience and influential mechanism during economic crises in China. Sci. Geogr. Sin..

[43] Ivanov D., Dolgui A. (2020). Viability of intertwined supply networks: Extending the supply chain resilience angles towards survivability. A position paper motivated by COVID-19 outbreak. Int. J. Prod. Res..

[44] Martin R., Sunley P., Gardiner B., Tyler P. (2016). How regions react to recessions: Resilience and the role of economic structure. Reg. Stud..

[45] Wang Z.X., Wei W. (2021). Regional economic resilience in China: Measurement and determinants. Reg. Stud..

[46] Lawreniuk S. (2020). Necrocapitalist networks: COVID-19 and the ‘dark side’ of economic geography. Dialogues Hum. Geogr..

[47] David L. (2018). Agency and resilience in the time of regional economic crisis. Eur. Plan. Stud..

[48] Manca A.R., Benczur P., Giovannini E. (2017). Building a scientific narrative towards a more resilient EU society. JRC Sci. Policy Rep..

[49] Mattana E., Smeets V., Warzynski F. (2020). Changing skill structure and Covid-19. Covid Econ..

[50] Wink R., Wink R. (2021). Introduction: Covid-19 pandemic as new challenge for regional resilience research?. Economic Resilience in Regions and Organisations.

[51] Harris J.L., Sunley P., Evenhuis E., Martin R., Pike A., Harris R. (2020). The Covid-19 crisis and manufacturing: How should national and local industrial strategies respond?. Local Econ..

[52] Li L.G., Zhang P.Y., Lo K., Liu W.X., Li J. (2020). The evolution of regional economic resilience in the old industrial bases in China: A case study of Liaoning Province, China. Chin. Geogr. Sci..

[53] Liu Y., Ji J.H., Zhang Y.F., Yang Y. (2020). Economic resilience and spatial divergence in the Guangdong-Hong Kong-Macao Greater Bay Area in China. Geogr. Res..

[54] Wu F., Liu G.J., Guo N.L., Li Z.H., Deng X.Z. (2021). The effects of COVID-19 epidemic on regional economy and industry in China. Acta Geogr. Sin..

[55] Vishnevskii D.S., Demyanenko A.N. (2012). Russian Far East: Macroeconomic zoning. Reg. Res. Russ..

[56] Martin R., Gardiner B. (2019). The resilience of cities to economic shocks: A tale of four recessions (and the challenge of Brexit). Pap. Reg. Sci..

[57] Wang J.F., Xu C.D. (2017). Geodetector: Principle and prospective. Acta Geogr. Sin..

[58] Brown L., Greenbaum R.T. (2017). The role of industrial diversity in economic resilience: An empirical examination across 35 years. Urban Stud..

[59] The Standard Map Service. http://bzdt.ch.mnr.gov.cn/.

[60] The Russian Federal State Statistics Service. http://www.gks.ru/.

[61] Gereffi G. (2020). What does the COVID-19 pandemic teach us about global value chains? The case of medical supplies. J. Int. Bus. Policy.

[62] Bryson J.R., Vanchan V. (2020). COVID-19 and alternative conceptualizations of value and risk in GPN research. Ijdschrift Econ. En Soc. Geograhie.

[63] Grundy-Warr C., Lin S. (2020). COVID-19 geopolitics: Silence and erasure in Cambodia and Myanmar in times of pandemic. Eurasian Geogr. Econ..

[64] Ezcurra R., Rios V. (2019). Quality of government and regional resilience in the European Union. Evidence from the great recession. Pap. Reg. Sci..

[65] Peng R.X., Liu T., Cao G.Z. (2021). Spatial pattern of urban economic resilience in eastern coastal China and industrial explanation. Geogr. Res..

[66] Klimanov V.V., Kazakova S.M., Mikhaylova A.A. (2019). Retrospective analysis of the resilience of Russian regions as socio-economic systems. Vopr. Ekon..

[67] Gong H.W., Hassink R., Tan J.T., Huang D. (2020). Regional resilience in times of a pandemic crisis: The case of COVID-19 in China. Tijdschr. Econ. En Soc. Geogr..

